# Sequential stamen maturation and movement in a protandrous herb: mechanisms increasing pollination efficiency and reducing sexual interference

**DOI:** 10.1093/aobpla/plx019

**Published:** 2017-05-25

**Authors:** Chang-Long Xiao, Hui Deng, Gan-Ju Xiang, Kadiori Edwin Luguba, You-Hao Guo, Chun-Feng Yang

**Affiliations:** 1 College of Life Sciences, Wuhan University, 430072 Wuhan, China; 2 Key Laboratory of Aquatic Botany and Watershed Ecology, Wuhan Botanical Garden, Chinese Academy of Sciences, 430074 Wuhan, China

**Keywords:** Movement herkogamy, pollen presentation theory, pollen transfer efficiency, pollination, sexual interference, stamen movement

## Abstract

Successive stamen movement directly controls pollen presentation schedules through sequential stamen maturation and changes the extent of herkogamy by altering the positions of sexual organs. However, the implications of such movements in terms of pollination are not well understood. Pollen presentation theory predicts that staggered pollen presentation should be favoured when plants are subject to diminishing returns on pollen transfer. Herkogamy on the other hand, has been interpreted as an adaptive trait that reduces sexual interference in hermaphrodite flowers. In this study, we conducted floral manipulations to determine the function of successive stamen movement in pollen transfer. By artificially manipulating the flowers to present two anthers simultaneously in the floral centre, we attempted to investigate whether changes in the anther presentation strategy affect pollen removal, deposition and the efficiency of pollinators. Compared with the natural treatment, the pollen transfer efficiency of halictid bees decreased significantly when the flowers were manipulated to present two anthers simultaneously. Although the presentation of two anthers simultaneously led to a similar pollen removal rate, there was a significant reduction in pollen deposition on neighbouring stigmas. To evaluate the effect of movement herkogamy on pollen export and deposition and seed set, the flowers were manipulated with or without the movement of stamen bending out from the floral centre. Pollen export decreased significantly when the central anther was moved away from the pistil, and pollen deposition and seed set declined significantly when the five spent anthers were retained on the pistil. Our study provides good support for the pollen presentation theory and provides direct experimental evidence that successive stamen movement could increase pollen transfer efficiency by sequential stamen maturation. Moreover, movement herkogamy promotes pollen export, deposition and seed set, and could therefore be regarded as an effective mechanism to reduce interference between male and female functions.

## Teaser

Successive stamen movement directly controls pollen presentation schedules through sequential stamen maturation and changes the extent of herkogamy by altering the positions of sexual organs. We conducted floral manipulations to determine the function of successive stamen movement in pollen transfer. Our study supports the pollen presentation theory and provides direct experimental evidence that successive stamen movement could increase pollen transfer efficiency by sequential stamen maturation. On the other hand, movement herkogamy promotes pollen export, deposition and seed set, and could therefore be regarded as an effective mechanism to reduce interference between male and female functions.

## Introduction

The majority of flowering plants rely on animals for pollination. The attraction of more floral visitors to participate in the process of pollen transfer and to improve pollination efficiency is one of the most pervasive driving forces in the evolution of floral traits (Faegri and Van der Pijl 1979; Bawa and Beach 1981; Real 1983). Due to the activities of pollinators, pollen loss during the process of pollen transfer is always very high, and normally <1 % of the pollen removed from the anthers can reach conspecific stigmas (Holsinger and Thomson 1994; Harder and Wilson 1998). Plants may reduce such uncertainty of successful pollen transport by adjusting the schedule of pollen presentation to the quantity and quality of pollinators (Lloyd and Yates 1982; Harder and Thomson 1989; Harder and Wilson 1994). Pollen presentation theory (PPT) suggests that plants with frequent and inefficient pollinators tend to present pollen gradually in doses, whereas simultaneous pollen presentation should be preferred when plants are pollinated by infrequent but efficient visitors (Harder and Thomson 1989; Harder and Wilson 1994; Li *et al.* 2014). Through either pollen packaging or dispensing mechanisms, plants can vary their pollen presentation schedules using various floral strategies, such as staggering the opening of anthers, flowers, or inflorescences or opening them simultaneously (Thomson and Barrett 1981; Harder and Wilson 1994; Castellanos *et al.* 2006).

Successive stamen movement, in which stamens move one by one or group by group to the floral centre for anther dehiscence, is a special kind of pollen packaging and dispensing pattern that presents pollen in small doses to floral visitors (Ren 2010; Ruan and da Silva 2011). It has been documented in several families, including Loasaceae, Celastraceae, Rutaceae and Tropaeolaceae (Weigend and Gottschling 2006; Henning and Weigend 2012; Ren and Tang 2012; Armbruster *et al.* 2014). Ren and Tang (2012) found that in *Ruta graveolens*, pollen removal was promoted through one-by-one stamen uplifting, and the interference between two consecutively dehisced anthers was reduced by one-by-one stamen falling back. Armbruster *et al.* (2014) recognized that stamen movement in *Parnassia epunctulata* could improve pollination accuracy by placing the anthers and stigma in the right place at the right time. In these studies, the authors observed that the flowers were frequently visited by a variety of insects. According to PPT, staggered pollen presentation should be favoured when pollinators are frequent and inefficient at pollen transfer. This rests on the critical assumption that there is a decelerating relationship (diminishing returns) between the amount of pollen removed in a visit and the amount of that pollen deposited onto stigmas (Harder and Thomson 1989; Thomson *et al.* 2000). However, it remains unclear whether and how this movement affects the relationship between pollen removal and deposition by the pollinators.

An additional feature of successive stamen movement is movement herkogamy, in which the distance between male and female organs changes with the repositioning of stamens. In hermaphrodite flowers, herkogamy is thought to function mainly to reduce interference between male and female functions (Lloyd and Webb 1986; Webb and Lloyd 1986; Barrett 2002a, 2002b; Medrano *et al.* 2005; Li *et al.* 2013). However, excessive herkogamy may greatly reduce the possibility that pollinators contact both sexual organs, while scanty herkogamy may induce physical interference as male and female organs block each other’s access to pollinators (Webb and Lloyd 1986; Barrett 2002b). For example, in *Mimulus aurantiacus*, pollinators exported twice as much pollen in flowers with closed stigmas compared to those with open stigmas because open stigmas physically interfered with pollen export (Fetscher 2001). To maximize male and/or female functions, the movement of fertile parts might be of evolutionary significance because the first functional organs could make way for the second and thus reduce the possibility of sexual interference (Barrett 2002b; Barrett 2003; Armbruster *et al.* 2014). Stamen movement provides us with an opportunity to detect whether and how this behaviour reduces sexual interference.

In this study, to assess the successive stamen movement in a protandrous herb *Parnassia wightiana* (Celastraceae) (Bremer *et al.* 2009), we investigated whether and how staggered pollen presentation affects the pollen transfer efficiency of the pollinators, i.e. the relationship between the amount of pollen removed in a visit and the amount deposited onto the stigma in a subsequent visit. By artificially manipulating the flower to simultaneously present two anthers in the floral centre, we attempted to investigate whether changes in anther presentation affect pollen removal, deposition and the efficiency of pollinators. In addition, the effect of movement herkogamy was evaluated by comparing pollen export, deposition and seed set when this movement was interrupted by floral manipulations. Based on field investigations, we aimed to address the following: (i) whether and how successive stamen movement affects pollen transfer efficiency and (ii) whether and how successive stamen movement provides movement herkogamy and reduces sexual interference.

## Methods

### Study species and site

The genus *Parnassia* consists of ∼70 species predominantly occurring in arctic and temperate regions of the Northern Hemisphere, with >30 of the species confined to China and the Himalayan region (Gu and Hultgard 2001). *Parnassia wightiana* is a hermaphroditic perennial herb distributed in various open and moist habitats, including riparian, grassy and shaded areas in valleys (Gu and Hultgard 2001). The plant is 18–24 cm in height and bears 5–15 flowers during the flowering period. Its white flowers have five green staminodes, occasionally with inconspicuous glands at a five-lobed apex with five fertile stamens aggregated around the pistil. Nectar was presumably produced at the inner surface of the base of the filaments around the ovary, but no measureable nectar was collected (also see Armbruster *et al.* 2014 for *P. epunctulata*).

Field investigations were conducted from July to August in 2013 and 2014 in the Qinling Mountains, Shaanxi, China. The plants grow on a large sloping meadow located in Taibai County, near Baoji City (34°02’41.83" N, 107°21’12.74" E, altitude 1523 m). The co-occurring species are *Trifolium pratense* (Leguminosae), *Saposhnikovia divaricata* (Umbelliferae), *Cirsium setosum* (Asteraceae), *Geranim dahuricum* (Geraniaceae), *Hypericum ascyron* (Guttiferae) and *Pedicularis resupinata* (Orobanchaceae).

### Pattern of stamen movement

To determine the full process of stamen movement, 30 tagged flowers were monitored from bud to the female phase in 2013. The positions and status of each anther were recorded with the help of a digital camera (Nikon D90) during the entire anthesis process. We quantified three events of stamen movement: (i) the time from flower opening to five immature stamens being below the level of the pistil; (ii) the process of each stamen extending and uplifting its anther to the top of the pistil; (iii) the process of each stamen bending outward to keep its anther away from the pistil. To further examine the pattern of movement herkogamy, we measured the distance between the moving anther and stigma using a vernier caliper every 2 h after flower opening.

### Breeding system

To identify the breeding system of *P. wightiana*, we conducted field experiments on 100 female phase flowers by imposing four pollination treatments. Flowers were randomly selected from different plants to reduce the possible effect of resource reallocation on fruit set, and were enclosed with fine-mesh polyester bags to exclude any pollinators before the artificial treatments. To test for potential autogamy, 25 flowers were caged to exclude any insects. In addition, 25 flowers were hand-pollinated with self-pollen grains from flowers of the same individual, and another 25 flowers were hand-pollinated with outcross pollen grains from multiple flowers of other individuals to test for any differences in seed production between selfing and outcrossing. The remaining 25 flowers without any treatment were exposed to open pollination as a natural control. Two weeks later, the fruits produced by these flowers were harvested, and the seeds and undeveloped ovules in each fruit were counted.

### Pollination

According to preliminary observations, we classified the floral visitors of *P. wightiana* into five functional groups: honey bees (Hymenoptera: Apidae), halictid bees (Hymenoptera: Halictidae), drone flies (Diptera: Syrphidae), blow flies (Diptera: Calliphoridae) and ants (Hymenoptera: Formicidae). Functional groups were used rather than species because the morphologies and foraging behaviours of members in a functional group are similar and also because functional groups are easier to recognize and more accurate to record (Fenster *et al.*, 2004). Insect observations were conducted from 0830 to 1130 and from 1430 to 1630 h on sunny days during peak flowering times. Three plots (1 × 1 m) were randomly established, each comprising 10–20 flowers. All three plots were observed daily for a period of 30 min. A total of 80 and 130 observation sessions were conducted in 2013 and 2014, respectively. In 20 of the sessions, we observed the foraging behaviours of all visitors and determined whether they touched any sexual organs.

The pollination system may vary from one population to the other, resulting in varying pollen transfer efficiency; this is measured as the proportion of pollen grains removed that were later deposited on a stigma (Inouye *et al.*, 1994). To quantify the pollen transfer efficiency, both pollen removal and pollen deposition per visit by each pollinator group were evaluated in the field populations. To avoid the possible effect of density, 105 male-phase flowers were randomly bagged with fine mesh at 0.5 m intervals. In addition to each male-phase flower, a neighbouring virgin female-phase flower was also bagged. When the central anther in a male-phase flower had fully dehisced, this flower and a neighbouring receptive female-phase flower were both exposed to insects for visitation. Once an insect had visited the male-phase flower and subsequently the neighbouring female-phase flower, the anther and stigma were collected separately and used to estimate the amount of pollen that remained in the anther and that was deposited on a stigma after a visit. An adjacent undehisced anther in the visited male-phase flower was collected to estimate pollen production.

In the laboratory, each collected anther was dissected and washed in 1 mL distilled water. Five 10 µL samples of suspension were used for counting the pollen after the solution had been stirred in a vortex mixer for 30 s. The pollen grains were counted under light microscopy at ×40 power (Nikon Eclipse E600). The number of pollen grains from all samples was averaged and multiplied by the dilution factor (100) to obtain the total number of pollen grains in an anther. Pollen removal per visit was calculated by subtracting the number of pollen grains remaining in the visited anther from the total pollen production. To assess pollen deposition, the stigmas were softened in 8 mol/L NaOH for 4 h and then mounted onto slides to count the number of pollen grains under a light microscope.

### The effect of anther presentation strategy on pollen transfer

In the natural male-phase flowers, only one anther releases pollen in the floral centre. To detect the potential effect of the anther presentation strategy on pollinator efficiency, we manipulated 55 male-phase flowers to simultaneously present two mature anthers in the floral centre **[see Supporting Information—Fig. S1A]**. When a flower was found to have an uplifted anther in the floral centre, the stamen was immobilized with a white thread to prevent subsequent deflexion. One end of the thread was bound to the joint between the filament and anther, and the other end was tethered to the pedicel through the space between two opposite petals, according to the manipulations piloted by Ren and Bu (2014). To minimize possible interference during insect visitation, we camouflaged the thread and petal and kept it outside the path of the floral visitors. The manipulations were conducted before anther dehiscence to prevent the shedding of pollen from the anthers. Then, the flowers were carefully bagged to prevent insect visitation. The next morning the next anther uplifted and moved close to the first, resulting in two mature anthers being presented simultaneously in the floral centre. A manipulated flower and a neighbouring previously bagged female-phase flower were both exposed to pollinators for visitation. The pollen removal and deposition per visit were then measured as before. Due to bad weather and the low visitation rates of honey bees and drone flies, we obtained only 25 pairs of pollen removal and deposition data for halictid bees. The counts of pollen grains removed and deposited were divided by the number of anthers per sample for subsequent statistical analysis. Pollen transfer efficiency was also calculated for the pollinators.

### The effect of herkogamy on pollen export and deposition

In natural male-phase flowers, the anthers are uplifted to the top of the pistil in the floral centre for pollen dispersal. To examine the effect of herkogamy on pollen export, we randomly selected 20 male-phase flowers and repositioned the uplifted anthers away from the pistil with a white thread before anther dehiscence **[see Supporting Information—Fig. S1B]**. One end of the thread was bound to the joint between the filament and anther, and the other end was tethered to the pedicel through the space between two homolateral petals. In addition to each manipulated flower, a natural male-phase flower was marked as a control. These flowers were exposed to pollinators after dehiscing. We specifically noted whether each visitor could contact the dehiscing anther. After 8 h, all anthers were collected and used to estimate the proportion of pollen export.

In natural female-phase flowers, all five dehisced anthers move away from the pistil by filament deflexion. To determine the effect of such movement on pollen deposition and seed set, 35 randomly selected flowers were manipulated without this movement. We halted deflexion by bending all five stamens together into the floral centre **[see Supporting Information—Fig. S1C]**. Before each stamen bent outwards, a thread was used to lash the stamen to the style. One end of the thread was bound to the joint between the filament and anther, and another end was tethered to the style. In addition to each manipulated flower, a natural female-phase flower was labelled as a corresponding natural control. After exposure to insects for 8 h, the stigmas of 15 manipulated and 15 control flowers were collected to estimate pollen deposition. Two weeks later, the remaining 20 manipulated and 20 control flowers were harvested to count the seeds and undeveloped ovules.

### Statistical analyses

For the pollination treatments, one-way ANOVA was used to detect the effects of the treatments on seed set. Seed set was square root-transformed to satisfy the assumption of homogeneity of variance. We also performed one-way ANOVA to test for differences in pollen removal and pollen transfer efficiency (with arcsine square root transformation) among insect groups. Because the transformed pollen deposition data did not satisfy the homogeneity of variance assumption, the Kruskal–Wallis test was used to analyse the differences among insect groups. When variables were revealed to be significantly different based on ANOVA and the Kruskal–Wallis test, Tukey’s HSD and the Mann–Whitey *U*-test were used as post hoc multiple comparison tests, respectively. In the anther presentation experiment, Student’s *t*-test was conducted to identify differences in pollen removal, deposition and transfer efficiency between the manipulated and natural treatments. In the herkogamy experiment, we also used Student’s *t*-test to identify differences in pollen export, pollen deposition and seed set between the manipulated and control treatments. All statistical analyses were performed in SPSS Statistics 20 (IBM SPSS, 2013).

## Results

### Pattern of stamen movement

**Figure 1.**
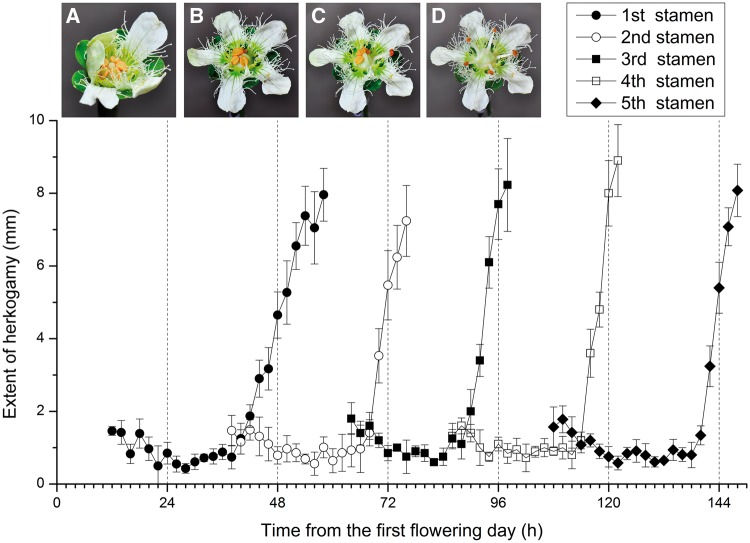
Details of successive stamen movement in *P. wightiana.* (A) All five immature stamens are below the pistil. (B) The first anther is on the top of the pistil. (C) The first anther is away from the pistil. (D) All stamens are away from the pistil. Dotted lines indicate the boundary between two successive days.

When the flower was at the budding phase, all five immature anthers were aggregated around the pistil with an anther-stigma distance of 1.67 ± 0.30 mm (mean ± SD, *N* = 30; Fig. 1A). On the first day of anthesis, the flowers opened slowly at ∼1000 h, and the first stamen began to uplift its anther at ∼1600 h (Fig. 1). It took 10–14 h for the anther to be moved to the position above the pistil (Fig. 1B). During this process, the distance between the moving anther and the stigma gradually decreased to 0.75 ± 0.22 mm (*N* = 30; Fig. 1). On the second day, the anther dehisced and released red pollen from 0800 to 1200 h. At ∼1600 h, the stamen began to bend outwards away from the pistil. Meanwhile, the second stamen began to uplift its anther. It took >12 h for the first stamen to finish deflexion and finally descend into the space between two homolateral petals (Fig. 1C). The distance between the first anther and the stigma increased to 8.23 ± 0.59 mm (*N* = 30; Fig. 1). The remaining stamens successively moved in the same pattern as the previous stamen. After the last stamen had bent outwards and clear of the pistil (Fig. 1D), the style elongated and diverged into three lobes, and the female phase followed [**see Supporting Information—Video S1** for a demonstration of the entire process of successive stamen movement]. 

### Breeding system

**Figure 2.**
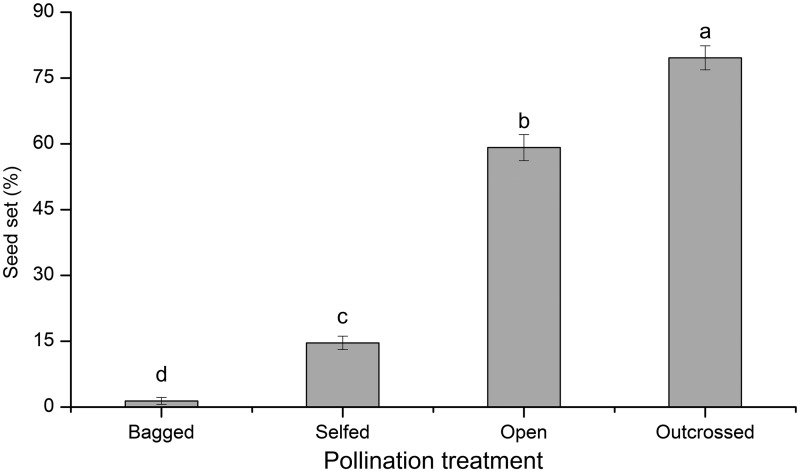
Breeding system of *P. wightiana*. Different letters above the bars show significant differences at *P *< 0.05 (one-way ANOVA). Bagged, bagged without any treatment, *N* = 18; Selfed, self-pollination, *N* = 25; Open, open-pollination, *N* = 16; Outcrossed, cross-pollination, *N* = 21.

On average, each *P. wightiana* flower produced 127,958 ± 9313 (mean ± SE, *N* = 30) pollen grains and 751 ± 28 ovules with a pollen:ovule ratio of 170. In the pollination treatments, a total of 80 fruits were harvested. One-way ANOVA showed that there was a significant effect of the treatments on seed set (*F*_3, 76_ = 314.904, *P *< 0.001, Fig. 2). The seed set of the self-pollinated flowers was 14.6 % ± 1.5 % (mean ± SE, *N* = 18, Fig. 2), which was significantly higher than that of bagged flowers (1.4 % ± 0.8 %, *N* = 16, Fig. 2) but lower than that of open-pollinated flowers (59 % ± 2.9 %, *N* = 25, Fig. 2). However, the plant was pollen-limited under natural conditions, as outcrossing yielded the highest seed set among these treatments (79.9 % ± 2.7 %, *N* = 21, Fig. 2).

### Pollination

**Figure 3.**
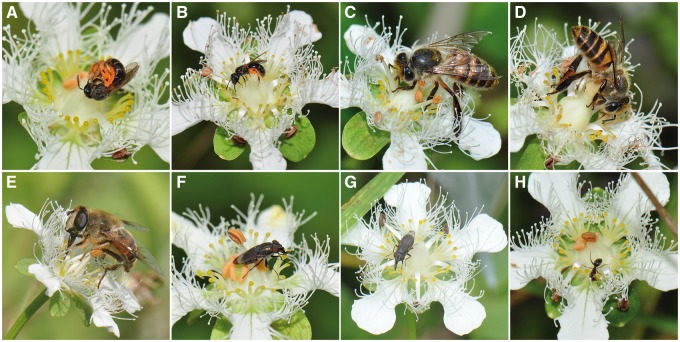
Diverse insects visiting flowers of *P. wightiana.* (A) A halictid bee collecting pollen grains from the central anther in a male-phase flower. (B) A halictid bee seeking nectar on a female-phase flower and touching the stigma. (C) A honey bee contacting the anther on a male-phase flower. (D) A honey bee obtaining nectar on a female-phase flower and touching the stigma. (E) A drone fly obtaining nectar on a male-phase flower and touching the anther. (F) A blow fly on a male-phase flower without contacting the dehiscing anther. (G) A blow fly on a female-phase flower without touching the stigma. (H) An ant sucking nectar without touching the central anther.

A total of 3182 and 3417 insect visits were recorded in 2013 and 2014, respectively (Table 1). Across the 2 years, the two most common visitors were halictid bees (59.98 %) and blow flies (27.57 %), with mean visitation rates of 1.87 ± 0.12 and 0.86 ± 0.06 visits per flower per hour, respectively (mean ± SE, *N* = 210 sessions). The remaining 12.44 % of the total visits were made by drone flies (0.19 ± 0.02 visits per flower per hour), honey bees (0.13 ± 0.04 visits per flower per hour) and ants (0.06 ± 0.02 visits per flower per hour). The five insect groups showed different foraging behaviours on the flowers. Halictid bees both collected pollen grains from the dehiscing anther (Fig. 3A) and sought nectar at the base of the staminodes (Fig. 3B). Honey bees (Fig. 3C and D) and drone flies (Fig. 3E) just obtained nectar from both male- and female-phase flowers. Halictid bees, honey bees and drone flies were seen to touch the anthers or stigmas in >70 % of visits (Table 1). Blow flies (Fig. 3F and G) and ants (Fig. 3H) often spent a long time sucking nectar, and blow flies sometimes also foraged for pollen from the dehiscing anther, but they rarely contacted the reproductive organs (Table 1).

**Table 1.** Insect visits to *Parnassia wightiana* flowers. A total of 40 and 65 h observations were carried out in 2013 and 2014, respectively. In 20 of the 30-min sessions (770 visits), we recorded whether each visitor was seen to contact the anther or stigma.

Visitor group	Number (%) of visits		Numbers (%) that touched	Reward
	2013	2014		
Halictid bees	1886 (59.27)	2072 (60.64)	363 (86)	Pollen, nectar
Blow flies	842 (26.46)	978 (28.62)	53 (26)	Pollen, nectar
Drone flies	228 (7.17)	180 (5.27)	61 (71)	Nectar
Honey bees	200 (6.28)	79 (2.31)	25 (75)	Nectar
Ants	26 (0.82)	108 (3.16)	3 (13)	Nectar
Total	3182	3417	506	

Our results showed that there were significant differences in the pollen removal among insect groups (*F*_2,__73_ = 8.340, *P *< 0.01; Table 2). Multiple comparisons demonstrated that halictid bees removed significantly more pollen per visit on average than both honey bees and drone flies. No significant difference was found between the amount of pollen removed by honey bees and drone flies. Pollen deposition per visit was also significantly different among the insect groups (Kruskal–Wallis, χ^2^ = 37.881, df = 2, *P *< 0.001; Table 2). Honey bees deposited significantly more pollen grains on stigmas than halictid bees, while halictid bees deposited a greater amount of pollen than drone flies. These pollinators showed significant differences in their pollen transfer efficiency when foraging on *P. wightiana* (*F*_2,__73_ = 30.453, *P *< 0.001; Table 2). The pollen transfer efficiency of honey bees was almost two times that of halictid bees and three times that of drone flies. We were unable to accurately estimate pollen removal and deposition by blow flies and ants because they rarely contacted the sexual organs.

**Table 2.** Pollen removal, pollen deposition and pollen transfer efficiency of pollinators of *Parnassia wightiana.* Values are means ± SEs. Sites with different letters among insect groups indicate significant differences (*P *< 0.05; Tukey’s HSD for removal and efficiency, Mann–Whitney *U*-test for deposition; *N* = 29 for halictid bees, *N* = 25 for drone flies and *N* = 22 for honey bees). Pollen removal is the number of pollen grains removed per visit in a male-phase flower. Pollen deposition is the number of pollen grains deposited on the stigma of a neighbouring female-phase flower. Pollen transfer efficiency was calculated as pollen deposition divided by pollen removal per visit.

Insect group	Halictid bees	Drone flies	Honey bees
Removal	23145 ± 698^a^	18904 ± 838^b^	20357 ± 786^b^
Deposition	82 ± 9^b^	37 ± 3^c^	130 ± 17^a^
Efficiency	0.36 ± 0.04%^b^	0.20 ± 0.01%^c^	0.60 ± 0.05%^a^

### The effect of anther presentation on pollinator efficiency

When the flowers were manipulated to present two mature anthers simultaneously, halictid bees removed 90.4 ± 2.94 % (mean ± SE, *N* = 25) of the pollen in the two anthers during a single visit, which was not significantly different from removal in the natural treatment (91.53 ± 2.39 %, *N* = 29, *P = *0.125, Student’s *t*-test). On the per-anther basis, there was also no measurable difference in pollen removal by halictid bees between the manipulated and natural treatments (*P = *0.516, Student’s *t*-test; Table 3). However, the subsequent pollen deposition to a neighbouring stigma by halictid bees was significantly lower in the manipulated treatment than in the natural treatment (*P < *0.05, Student’s *t*-test; Table 3). Consequently, the pollen transfer efficiency of halictid bees was significantly lower (*P < *0.001, Student’s *t*-test; Table 3).

**Table 3.** The comparisons of pollen removal, pollen deposition and pollen transfer efficiency of halictid bees between manipulated and natural treatments in *Parnassia wightiana.* Values are means ± SEs. Sites with different letters indicate significant differences (Student’s *t*-test, *P *< 0.05, *N*_mani_ = 25, *N*_natu_ = 29).

Treatments	Removal	Deposition	Efficiency
Manipulated	22459 ± 787^a^	55 ± 7^a^	0.24 ± 0.02%^a^
Natural	23145 ± 698^a^	82 ± 9^b^	0.36 ± 0.04%^b^

### The effect of herkogamy on pollen export and deposition

**Figure 4.**
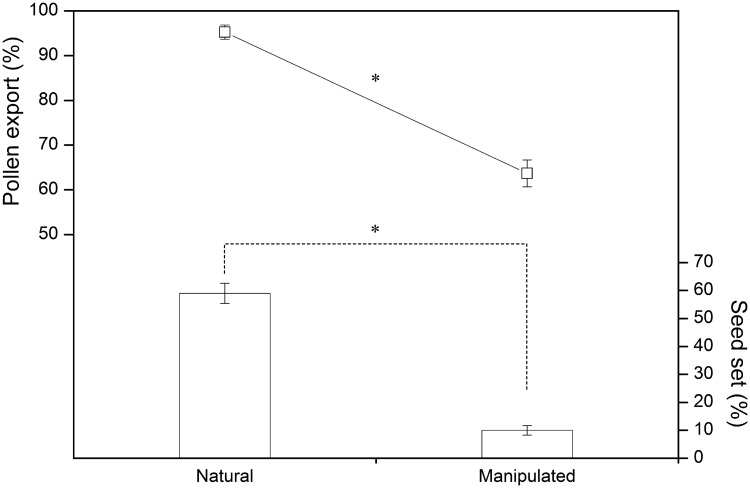
The effect of herkogamy treatments on pollen export and seed set. (‘*’ *P *< 0.01, Student’s *t*-test, *N*_natu_ = *N*_mani_ = 20).

For the natural male-phase flowers, 69 % (281 out of 405 visits) of the insects touched the central anther, and 95.4 % ± 0.7 % (mean ± SE, *N* = 20; Fig. 4) of the pollen in the anther was exported. When the central anther was moved away from the pistil for pollen dispersal, only 24 % (92 out of 390 visits) of insects contacted the anther. The percentage of pollen export decreased significantly as a result (63.5 % ± 2.9 %, *P *< 0.001, Student’s *t*-test, *N* = 20; Fig. 4).

When all five stamens of the female-phase flowers were retained in the floral centre, the anthers and stigma were sufficiently close to each other. After a period of 8 h, only 40 ± 3 (*N* = 15) pollen grains had been deposited on individual stigmas in the manipulated treatment, which was significantly fewer than those deposited on the natural flower stigmas (234 ± 17, *P *< 0.001, Student’s *t*-test, *N* = 15). Seed set of the manipulated flowers was 10.2 % ± 1.2 % (*N* = 20), which was significantly lower than that of the control flowers (60.9 % ± 3.6 %, *P *< 0.001, Student’s *t*-test, *N* = 20; Fig. 4).

## Discussion

The evaluation of the breeding system indicates that *P. wightiana* should be partly self-compatible and is pollen-limited under natural conditions. The insects visiting the flowers of *P. wightiana* were diverse and numerous, but only honey bees, halictid bees and drone flies could effectively deposit pollen grains on stigmas. Blow flies and ants were often regarded as nectar or pollen consumers, as they rarely contacted the sexual organs of the flowers (Goulson 1999; Medrano *et al.* 2005). The relative importance of floral visitors to plant reproductive success depends on both the visitation frequency and pollen deposition ability of the pollinators (Thomson 2003; Fenster *et al.* 2004). In *P. wightiana*, halictid bees were the most important pollinators because ∼60 % of the total visits were made by halictid bees, and they deposited adequate pollen onto the stigmas. Honey bees and drone flies contributed little to pollination because of their quite low visitation frequency, although they were effective in pollen removal and deposition. As a consequence, we conclude that the *P. wightiana* population is largely reliant on halictid bees for pollination.

Stamen movement in *P. wightiana* is a highly choreographed process that involves presenting one fresh anther on the top of the pistil for pollen dispersal in the morning and then casting the dehisced anther clear of the pistil in the afternoon each day over a period of 5 days. As the stamen moves, the distance between the moving anther and stigma first decreases and then increases. Such stamen movement is similar to that observed in other *Parnassia* species, i.e. *P. palustris* (Sandvik and Totland 2003; Ren and Bu 2014) and *P. epunctulata* (Armbruster *et al.* 2014). Successive stamen movement in *P. wightiana* is thus characterized by two features: staggered stamen maturation and movement herkogamy.

Pollen presentation theory suggests that selection to maintain staggered pollen presentation should be high in cases involving pollinators that are abundant and inefficient at pollen transfer (Thomson 2003; Castellanos *et al.* 2006; Li *et al.* 2014). In our study, halictid bees were both frequent and relatively inefficient pollinators compared with honey bees, as they made 60 % of the total visits and subsequently deposited only 0.36 % of the removed pollen onto a stigma. Specifically, when the flowers were manipulated to present two anthers simultaneously, the pollen transfer efficiency of halictid bees declined significantly to 0.24 %. This is because although the presentation of two anthers simultaneously in the manipulation experiment led to a similar pollen removal rate it resulted in a significant reduction in the pollen deposition to neighbouring stigmas. Although the pollen transfer efficiency might be underestimated in light of the trials only tracking pollen deposition on the first receptive flower, the measurements should still be informative to demonstrate the diminishing returns on pollen transfer. It has been suggested that animal pollination probably results in diminishing returns through diverse mechanisms, including a limited capacity to carry pollen, pollinator grooming, layering of pollen on the pollinator and local mate competition (Harder and Wilson 1994; Harder and Johnson 2008). In our observations, halictid bees actively scratched a large amount of pollen from anthers into their corbiculae and groomed pollen grains off the stigma-contacting surfaces of their bodies. These pollen grains have little or no chance of being deposited on a stigma (Rademaker *et al.* 1997). This could be the main reason for the decline in the pollen transfer efficiency of halictid bees in the manipulated treatment. In some other studies, researchers have also indicated that the pollen available for pollination would rapidly decline and that pollination efficiency would decrease if plants were visited by pollen-collecting pollinators (Castellanos *et al.* 2003; Young *et al.* 2007). Notably, under such circumstances, sequential stamen maturation (staggered pollen presentation) could be a mechanism to increase the pollen transfer efficiency of pollinators in order to alleviate the effect of diminishing returns on pollen transfer. Moreover, sequential stamen maturation prolonged the duration of pollen dispersal time. A prolonged duration of pollen dispersal could distribute pollen onto more individual pollinators, which may also be beneficial for pollen transfer.

A recent study in the *Parnassia* species *P. palustris* demonstrated that one-by-one stamen movement could decrease ‘anther-anther’ interference by reducing the amount of pollen wasted in anthers (Ren and Bu 2014). However, such interference was not found in our study because the pollen removal rate was similar when one or two anthers were presented at once. This could be attributed to the lack of pollen foraging insects as well as the manipulation method used. It would be instructive to distinguish the differences in the evolutionary significance of stamen movement among closely related species when they occur in habitats with different pollination environments.

The anthers of *P. wightiana* flowers differed in position between the male and female phases due to stamen movement, which significantly promoted pollen export in the male phase and reduced sexual interference in the female phase. In male-phase flowers, we demonstrated that pollen export declined when the anther was moved away from the pistil because most floral visitors were seeking nectar in the floral centre without touching the anther. Uplifting the anther to a position above the pistil in the floral centre for pollen dispersal could maximize pollen export by increasing the probability of contact between the anther and pollinators (Schlindwein and Wittmann 1997). Moreover, the existence of dichogamy reduces the risk of self-pollination because the stigma is receptive only after all anthers have been cast away from the pistil. In the female phase, seed set significantly decreased when the five stamens were confined around the pistil because the anthers obstructed the deposition of pollen onto the stigma. This corresponds with the fact that male–female conflict occurs when sexual organs block each other’s access to pollinators (Fetscher 2001; Barrett 2002b; Armbruster *et al.* 2014). Therefore, the trait of all stamens bending away from the pistil reflects an adaption to avoid physical interference from the dehisced anthers to the receptive stigma.

## Conclusions

We provided direct experimental evidence that successive stamen movement in *Parnassia wightiana* could increase pollen transfer efficiency by sequential stamen maturation in the presence of frequent and inefficient pollinators, therefore supporting pollen presentation theory. Moreover, this stamen movement helped to form a movement herkogamy mechanism, which reflects an adaption to reduce interference between male and female functions. However, stamen movement should be under various kinds of environmental selective pressures. Detailed studies are needed to investigate the physiological basis of stamen movement under a systematic framework, which may be helpful in better understanding the evolutionary pathway of floral movements.

## Sources of Funding

This study was supported by the National Natural Science Foundation of China (31170214 to YHG and 31370263 and 31070206 to CFY).

## Contributions by the Authors

C.L.X., Y.H.G. and C.F.Y. conceived the study and C.L.X., H.D. and G.J.X. carried out the investigations; C.L.X. analyzed the data; C.L.X., C.F.Y. and K.E.L. wrote the manuscript.

## Conflict of Interest Statement 

None declared.

## Supplementary Material

Supplementary Data

## Supporting Information

The following additional information is available in the online version of this article—

**Figure S1.** Floral manipulations on the flowers of *P. wightiana.* (A) A male-phase flower is manipulated to present two anthers simultaneously. (B) An uplifted anther is moved away from the floral centre and immobilized by adhering to a sepal on a male-phase flower. (C) All five stamens are confined together in the floral centre of a female-phase flower. Manipulated anthers are marked with arrows.

**Video S1.** The whole process of successive stamen movement in *P. wightiana.*
