# Supplementary material for: Sequential stamen maturation and movement in a protandrous herb: mechanisms increasing pollination efficiency and reducing sexual interference
Source: AoB Plants. 2017 May 25;9(3):plx019. doi: 10.1093/aobpla/plx019 (PMC5499893; doi:10.1093/aobpla/plx019)

**Figure S1.** Floral manipulations on the flowers of *P. wightiana.* (A) A male-phase flower is manipulated to present two anthers simultaneously. (B) An uplifted anther is moved away from the floral centre and immobilized by adhering to a sepal on a male-phase flower. (C) All five stamens are confined together in the floral centre of a female-phase flower. Manipulated anthers are marked with arrows.


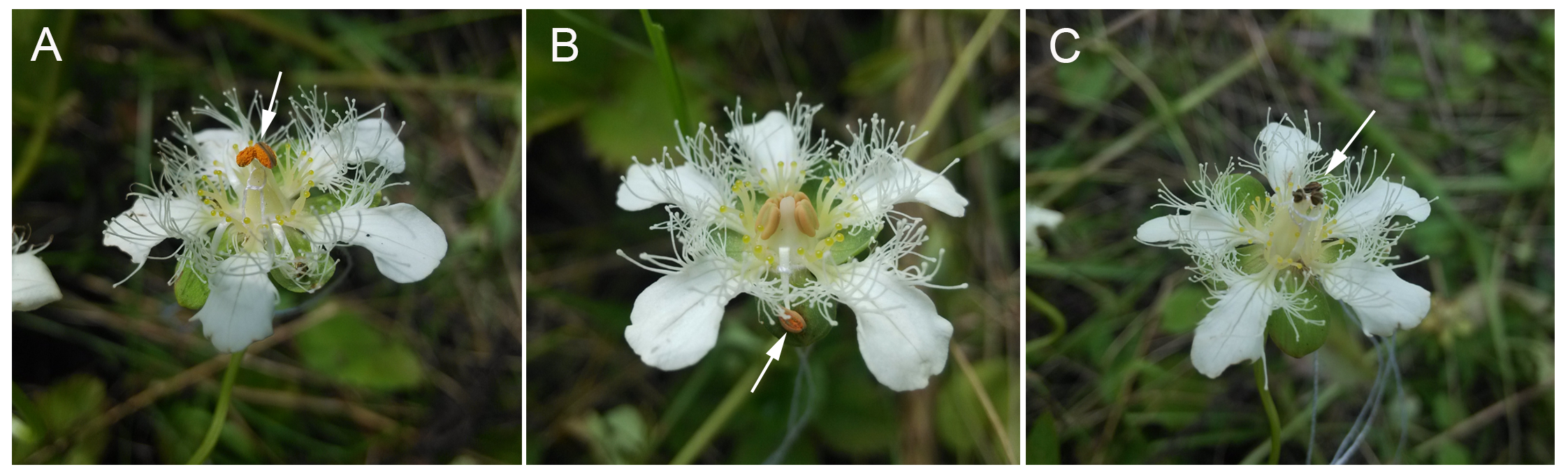

Supplement: Supplementary Data [file plx019_supp.zip › Figure S1.docx]
